# Alcohol addiction complicated with comorbid amnestic disorders: The search for innovative approaches to treatment

**DOI:** 10.1192/j.eurpsy.2021.1500

**Published:** 2021-08-13

**Authors:** I. Sosin, Y. Babenko, O. Sergienko, G. Mysko, O. Honcharova, I. Lisova

**Affiliations:** 1 Narcology Department, Kharkiv, Kharkiv Medical Academy of Postgraduate Education, Kharkiv, Ukraine; 2 Narcology Department, Kharkiv Medical Academy of Postgraduate Education, Kharkiv, Ukraine

**Keywords:** Alcohol addiction, Cognitive disorders, Amnesia, Treatment

## Abstract

**Introduction:**

Alcohol amnesia and palimpsests belong to understudied areas in addictology concerning the pathogenesis, risk factors, and development of effective integrated, targeted modalities of therapy, prevention, and after-treatment care.

**Objectives:**

Development of a new integrated, pathogenetically grounded approach to the emergency and routine therapy for immediate and long-term consequences of amnestic alcohol intoxication.

**Methods:**

Modern complex clinical-psychopathological, pathopsychological, laboratory, electrophysiological, biochemical examination; method of analogues and prototype analytical examination.

**Results:**

Integrated anti-amnestic pharmacotherapeutic triad: Noobut IC (Phenibut) orally, before meals, for 6 days, twice a day: 250 mg in the morning, 500 mg at night, within days 7-14 250 mg twice: in the morning and at night; Vitaxon 2.0 ml daily, intramuscularly, No10 totally; ozone therapy for 10 days (ozone dissolved in olive oil, 6 mg/100 ml concentration), 5 ml orally 3 times a day. Complex therapy is concurrent with synergistic psychotherapeutic potentiation. Supportive anti-relapse prevention of alcohol-induced amnesia, palimpsests with Noobut IC: 1 tablet (250 mg) orally in the morning for 2 months. The pathogenetic support of the pharmacotherapeutic triad in treatment for alcohol addiction, comorbid with amnestic disorders, is pathogenetically focused on pharmacological properties of each component of the triad and their potentiating effects, involving most pathogenetic mechanisms of this disease.

**Conclusions:**

Relieving and prophylactic efficacy of the proposed pharmacological triad (Noobut, Vitaxon, ozone and concurrent psychotherapeutic potentiation) is proven by the statistical reliability method and illustrated by clinical examples of patient-specific research.

